# Short tau inversion recovery (STIR) after intravenous contrast agent administration obscures bone marrow edema-like signal on forefoot MRI

**DOI:** 10.1007/s00256-021-03852-2

**Published:** 2021-07-13

**Authors:** Tim Fischer, Yassir El Baz, Stephan Waelti, Simon Wildermuth, Sebastian Leschka, Sabine Güsewell, Tobias Johannes Dietrich

**Affiliations:** 1 Division of Radiology and Nuclear Medicine, Kantonsspital St. Gallen, St. Gallen, Switzerland; 2 Faculty of Medicine, University of Zurich, Zurich, Switzerland; 3 Clinical Trials Unit, Kantonsspital St. Gallen, St. Gallen, Switzerland

**Keywords:** Magnetic resonance imaging, Foot, Contrast media, Bone marrow edema-like signal

## Abstract

**Objective:**

Short tau or short TI inversion recovery (STIR) MRI sequences are considered a robust fat suppression technique. However, STIR also suppresses signals from other tissues with similar T1 relaxation times. This study investigates the in vivo effect of intravenous gadolinium-based T1-shortening contrast agent on STIR signal.

**Materials and methods:**

Institutional board approval and informed consent was obtained. MRI examinations (1.5-T or 3-T) of 31 prospectively included patients were analyzed by two readers. Signal intensity of degenerative bone marrow edema-like signal at the Lisfranc joint on precontrast STIR images and on STIR images acquired after intravenous contrast agent administration (gadoteric acid, gadolinium: 0.5 mmol/ml, 15 ml) was measured. The medial cuneiform bone without observable bone marrow edema-like signal was considered a healthy tissue and served as a reference. Relative changes in signal intensity between precontrast and postcontrast images were calculated for the two tissues. Wilcoxon signed-rank test served for statistical analyses.

**Results:**

In bone marrow edema-like signal, both readers observed a median signal change of -35% (interquartile range (IQR) 24) and -34% (IQR 21), respectively, on postcontrast STIR images compared to precontrast STIR. In healthy tissue, the signal remained constant on postcontrast STIR images (median change -2%, IQR 15, and 0%, IQR 17) respectively. For both readers, postcontrast signal change in bone marrow edema-like signal differed from that in healthy tissue (*p* < 0.001).

**Conclusion:**

Intravenous gadolinium-based contrast agent causes a significant reduction of signal intensity in bone marrow edema-like signal on routine STIR images. Thus, pathological MRI findings may be obscured.

## Introduction

Besides conventional T1-weighted and T2 weighted imaging, short tau inversion recovery or short TI inversion recovery (STIR) MR sequences with a short inversion time (approximately 140 ms at 1.5-T) are used as a fat suppression technique. However, fat suppression with STIR is based on short T1 relaxation rates and therefore is not tissue specific. The signal from any tissue with a short T1, similar to that of fat, is nulled. An appropriate degree of T1 shortening due to accumulation of paramagnetic contrast agent in tissue may decrease the signal intensity on STIR sequence MR images [1, 2].

In vitro studies have shown that the signal in STIR after administration of gadolinium-based contrast agent is concentration-dependent. Based on T1 shortening effect, gadolinium concentrations between approximately 0.5 and 1.0 mmol per liter result in very low or even neutralized signal intensity on STIR images [3, 4].

This effect of gadolinium-based contrast agent as a potential pitfall on the detection of bone-marrow like edema-like signal on postcontrast STIR images is well-known among experienced radiologists in clinical routine; however, it has not been shown in vivo in any English peer-reviewed original article. Thus, it was hypothesized that bone marrow edema-like signal may be systematically obscured on postcontrast STIR images. The aim of this prospective study was to evaluate the in vivo effect of intravenous gadolinium-based contrast agent administration on bone marrow edema-like signal [5] on STIR images.

## Materials and methods

At our institution, intravenous contrast agent administration is frequently indicated for MRI examinations of the forefoot. Based on our experience, degeneration-related bone marrow edema-like signal findings at the Lisfranc joint are common. Thus, the Lisfranc joint was chosen to evaluate the in vivo effect of intravenous gadolinium-based contrast agent administration on bone marrow edema-like signal [5] on STIR images.

### Patient selection

Institutional board review (IRB) approval and patients’ informed consent were obtained (ID: 2020–00,283). In this prospective study, a total of 60 patients were consecutively included between April and September 2020. All patients referred to our institution for forefoot MRI with indication for intravenous gadolinium-based contrast agent administration were eligible for this study: At our institution, contrast-enhanced MR images are standard in forefoot protocols for the evaluation of osteomyelitis, Morton neuroma, neoplasm, vascular malformations including MR angiography, and rheumatic disorders. Patients were excluded from the study if there were contraindications for MRI or administration of intravenous contrast agent, age below 18-years, no obtainable patient’s informed consent, or severe motion artifact.

One experienced radiologist not involved in further imaging readout screened the precontrast STIR sequence of all eligible patients for the presence of visible bone marrow edema-like signal adjacent to the Lisfranc joint (distal cuneiform, distal cuboid, or proximal metatarsal bone). If no visible bone marrow edema-like signal was present or diffuse signal alteration consistent with disease other than degeneration in the Lisfranc joint, patients were excluded from the study.

### Imaging

MR images were acquired with 3-T Skyra or 1.5-T Avanto-fit MRI scanner (Siemens Healthineers). An example of the standard scanning parameters at 3-T MRI is given in Table 1. For this study, in addition to our institutional routine forefoot scanning protocol, a second STIR sequence after intravenous contrast agent administration was acquired with the same orientation and scanning parameter as in the precontrast sequence with prior recalibration. Gadoteric acid (Dotarem, Guerbet AG) with a gadolinium concentration of 0.5 mmol/ml and 15 ml volume was injected intravenously. STIR image acquisition started one minute after contrast agent administration.

**Table 1:** Detailed 3 T MRI forefoot protocol

	T1-w	T1-w	PD-w fs	STIR	Postcontrast T1-w fs
Orientation	Sagittal	Transversal	Transversal	Sagittal	Sagittal
FOV (mm × mm)	150 × 150	120 × 120	120 × 120	150 × 150	150** × **150
Matrix	358 × 448	230 × 384	200 × 320	256 × 320	288 × 320
TA (min:sec)	3:17	1:47	2:04	2:29	2:44
Number of slices	27	45	35	27	27
Slice thickness (mm)	3	4	4	3	3
Spacing between slices (mm)	3.3	4.4	4.4	3.3	3.3
TE (ms)	15	10	31	32	13
TR (ms)	780	720	3400	4210	765
ETL	3	5	6	8	6
TI (ms)				190	

*T1-w* T1-weighted, *PD-w fs* proton density-weighted, *STIR* short tau inversion recovery or short TI inversion recovery, *fs* fat-suppressed, *FOV* field of view, *TA* acquisition time, *TE* echo time, *TR* repetition time, *ETL* echo train length, *TI* time of inversion. TI is 150 ms for 1.5-T MRI scanner

### Image evaluation

Blinded and randomized image evaluation of all 31 patients was performed independently by a fellowship-trained musculoskeletal radiologist and a general radiologist with 5- and 6-years of experience in musculoskeletal radiology after a training of five cases that were not included in the study population. The two STIR sequences were randomly displayed on the left and on the right side of the screen to both readers blinded to the information which particular MR images were acquired before and after contrast agent administration. Evaluation was started on the STIR image displayed on the left screen. For each ray, the bone marrow of the bones adjacent to the Lisfranc joint was evaluated for signal alteration, consistent with bone marrow edema-like signal due to degenerative changes. For each digit, the bones adjacent to the Lisfranc joint were individually evaluated, resulting in a potential maximum of five proximal and five distal, in total ten measurements. As described above, bone marrow edema-like signal was required in at least one anatomical location. Means of repeated measurements were used for data analysis. For standardization, other skeletal regions were not included, even if bone marrow edema-like signal was visible.

Measurements were performed by using our PACS system toolbox (Impax, Agfa HealthCare NV). A circular region of interest (ROI) was drawn to include as much bone marrow edema-like signal as possible on the left screen. Bone marrow with normal signal was avoided. Average signal intensity values were read. Cystic changes were avoided.

A similar measurement was performed on the STIR sequence images displayed on the right screen using the automated ROI transfer option from the PACS toolbox. For comparison, the medial cuneiform bone was measured in a region with no visually perceptible bone marrow edema-like signal in both displayed sequences. The obtained SI values of the medial cuneiform bone were considered to represent healthy tissue. When precontrast and postcontrast measurements revealed the same SI value, this was referred to as unchanged signal. ROI sizes were recorded from reader 2.

### Statistics

Statistical analysis was performed with the statistical software R, version 4.0.2 (R Foundation for Statistical Computing (2020), Vienna, Austria). Interobserver agreement of average signal intensity was assessed by the two-way random effects intraclass correlation coefficient (ICC). ICC values > 0.75 were considered good agreement and > 0.9 as very good [6].

The effect of gadolinium-based contrast agent on STIR imaging was assessed by calculating the relative (%) change in signal intensity between precontrast and postcontrast images, separately for bone marrow edema-like signal (based on means of repeated measurements) and for healthy tissue. Results were summarized by reporting the median and interquartile range (IQR) of relative signal changes and the frequency of positive and negative changes. Correlations between signal intensities on precontrast and postcontrast images were evaluated for each tissue type with Pearson correlation coefficients to assess the consistency of signal changes. Relative signal changes in bone marrow edema-like signal were compared with those in healthy tissue using Wilcoxon signed-rank tests. In all tests, a p value of < 0.05 was considered to represent statistical significance.

## Results

**Fig. 1:**
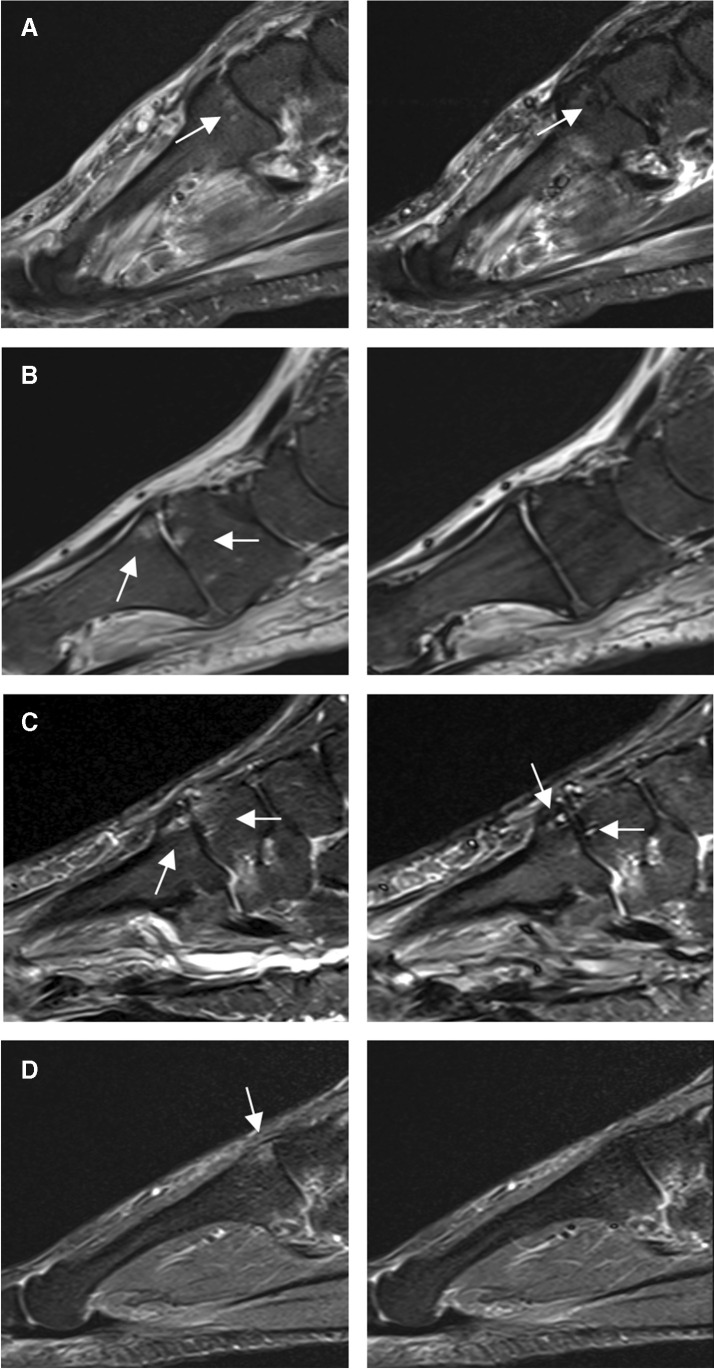
Obscured bone marrow edema-like signal on postcontrast STIR images. Precontrast STIR images of four different patients (**A** to **D**) with bone marrow edema-like signal at the Lisfranc joint (left) show signal suppression on postcontrast STIR images (right). **A** 1.5-T MRI of an 84-year-old male patient shows bone marrow edema-like signal at the base of the second metatarsal bone on precontrast STIR (left, white arrow). The signal intensity of bone marrow edema-like signal is reduced even below the adjacent normal appearing bone on the corresponding postcontrast STIR image (right, white arrow). **B** 1.5-T MRI of a 94-year-old male patient with bone marrow edema-like signal on both sides of the Lisfranc joint of the first ray (left, white arrow) on precontrast STIR with complete suppression of the signal on postcontrast STIR (right). **C** 3-T MRI of a 69-year-old female patient shows bone marrow edema-like signal with cystic changes at the Lisfranc joint of the second ray on precontrast STIR (left, white arrow) with nearly complete signal suppression on postcontrast STIR (right). Note the persisting high signal of the cystic changes on the postcontrast STIR image (white arrow). **D** 1.5-T MRI of a 37-year-old female patient with bone marrow edema-like signal at the base of the second metatarsal bone on precontrast STIR (left, white arrow) with complete signal suppression on the postcontrast STIR image (right)

Out of the *n* = 60 initially included patients, *n* = 29 did not reveal bone marrow edema-like signal MRI findings at the Lisfranc joint and were excluded from the analysis of this study. Mean age was 68 years, standard deviation (SD) ± 19.5 years, median 67.7 years, IQR 24.8 years, range 23–99 years, 17 males, and 14 females. Imaging examples of precontrast STIR and postcontrast STIR are given in Fig. 1.

### Interobserver agreement

Interobserver agreement (absolute agreement) between both readers for edema measurements was good in precontrast STIR images (ICC 0.80, 95% confidence interval (CI) 0.57 to 0.90) and postcontrast STIR (ICC 0.84, 95% CI 0.51 to 0.93). Interobserver agreement for measurements in healthy tissue was very good on precontrast STIR (ICC 0.98, 95% CI 0.96 to 0.99) and postcontrast STIR images (ICC 0.97, 95% CI 0.95 to 0.99).

### Average signal intensity measurements precontrast and postcontrast

**Fig. 2:**
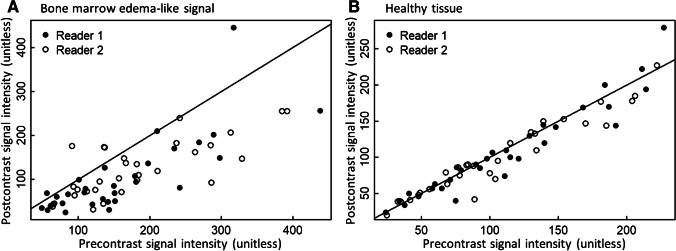
Correlation of average signal intensity of **A** bone marrow edema-like signal and **B** healthy tissue on precontrast STIR and postcontrast STIR images. Measured average signal intensity (SI) on precontrast STIR and postcontrast STIR images in **A** bone marrow edema-like signal and **B** healthy tissue for both readers. Reader 1 is represented by black dots; reader 2 is represented by white circles. The diagonal line indicates the 1:1 relationship expected if there is no change in signal intensity after contrast agent administration

Drawn ROIs in bone marrow edema-like signal had a mean diameter of 4.8 ± 1.5 mm, range 4 to 9 mm. Drawn ROIs in healthy tissue had a mean diameter of 8.5 ± 2.3 mm, range 5 to 15 mm. Results of average signal intensity measurements in bone marrow edema-like signal are given in Fig. 2. Obtained signal intensity values were highly variable. For measurements in bone marrow edema-like signal on STIR images, the correlation coefficient before and after contrast agent administration was 0.79 (95% CI 0.60 to 0.89) for reader 1 and 0.76 (95% CI 0.55 to 0.88) for reader 2. In healthy tissue, the correlation coefficient before and after contrast agent administration was 0.95 (95% CI 0.91 to 0.98) for reader 1 and 0.94 (95% CI 0.88 to 0.97) for reader 2.

### Relative changes in signal intensity after contrast agent administration

A decreased signal intensity was observed in bone marrow edema-like signal in the majority of patients on postcontrast STIR images compared to precontrast STIR images: Measurement by reader 1 observed a decreased signal intensity in 90% (n = 28/31) of patients, whereas an increased signal intensity was observed in 7% (n = 2/31) of patients on postcontrast STIR images compared to precontrast STIR images. In 3% (n = 1/31) of patients, signal intensity remained unchanged. The corresponding values for reader 2 was a decreased signal intensity in 87% (n = 27/31) and an increased signal intensity in 13% (n = 4/31) of patients on postcontrast STIR images compared to precontrast STIR images.

For measurements in apparently healthy tissue of the medial cuneiform bone, postcontrast changes in signal intensity values were more symmetrically distributed around zero (no change): Reader 1 observed a decreased signal intensity in 51% (n = 16/31) of patients, an increased signal intensity in 39% (n = 12/31) of patients, and an unchanged signal intensity in 10% (n = 3/31) of patients on postcontrast STIR compared to precontrast STIR images.

The corresponding values for reader 2 was a decreased signal intensity in 48% (n = 15/31) of patients, an increased signal intensity in 39% (n = 12/31) of patients, and an unchanged signal intensity in 13% (n = 4/31) of patients on postcontrast STIR compared to precontrast STIR images.

**Fig. 3:**
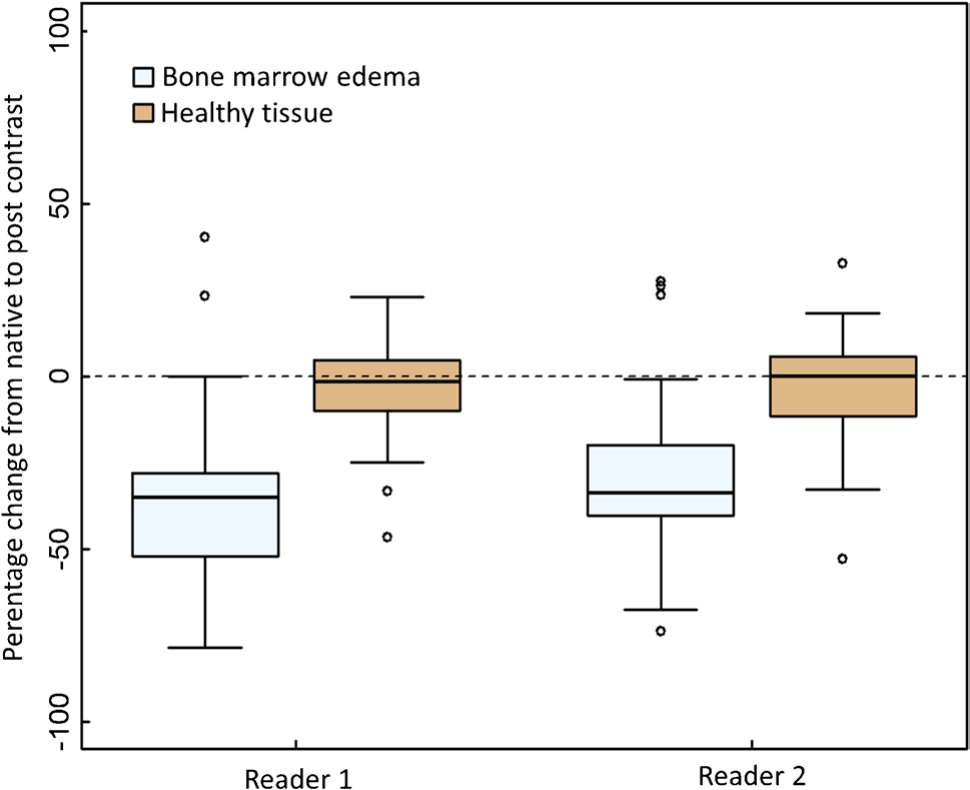
Relative signal change as percentage values (%) between precontrast STIR and postcontrast STIR images. Boxplots of relative signal change (% of SI on precontrast STIR images) between measured signal intensity (SI) on precontrast STIR images compared to postcontrast STIR for bone marrow edema-like signal and healthy tissue for both readers. Horizontal line indicates median; lower and upper edge of box indicates first and third quartile. Whiskers represent minimum and maximum, outliers excluded. Outliers (> 1.5 IQR) are indicated by circles. Relative signal change (precontrast to postcontrast) differed significantly between bone marrow edema-like signal and healthy tissue for both readers (p < 0.001, Wilcoxon signed-rank test)

The distributions of relative signal intensity changes after intravenous contrast for bone marrow edema-like signal and healthy tissue are given in Fig. 3.

For reader 1, median relative signal change on postcontrast STIR compared to precontrast STIR images for bone marrow edema-like signal measurements was -35% (IQR 24, 1st quartile -52%, 3rd quartile -28%), whereas median relative signal change in healthy tissue was -2% (IQR 15, 1st quartile -10%, 3rd quartile 5%).

For reader 2, median relative signal change on postcontrast STIR compared to precontrast STIR images for bone marrow edema-like signal measurements was -34% (IQR 21, 1st quartile -40%, 3rd quartile -20%), whereas median relative signal change in healthy tissue was 0% (IQR: 17, 1st quartile -12%, 3rd quartile 6%).

For both readers, relative signal changes on postcontrast STIR compared to precontrast STIR images in bone marrow edema-like signal differed significantly from those in healthy tissue (Wilcoxon signed-rank test, p < 0.001).

## Discussion

This study showed that signal intensity of degenerative bone marrow edema-like signal on postcontrast STIR images is significantly reduced compared to signal intensity on precontrast STIR images. Thus, detection of pathological tissue is more challenging and is unreliable on postcontrast STIR images compared to precontrast STIR images.

The STIR sequence uses a short inversion time to null T1 values, typical for fat which results in a fat suppressed imaging. In contrast to other fat suppression techniques, STIR signal is usually homogenous and less susceptible to magnetic field inhomogeneities [1, 2, 7]. Signal suppression is not tissue specific; thus, all tissues with similar T1 values in the range of fat, such as gadolinium containing tissue or proteinaceous materials, will be suppressed [1].

In MR imaging, an absolute signal intensity calibration does not exist as opposed to the Hounsfield units scale in computed tomography. Thus, signal intensity values may vary depending on software and hardware settings. Moreover, variation of signal intensity value measurements may be attributed to low pixel counts of the ROI, allowing random effects to introduce appreciable variance. Thus, MRI system noise contributes fractionally more to a pixel’s signal intensity in low or very low signal intensity measurements.

In this study, pre- and postcontrast STIR image acquisitions were within the same exam separated by approximately four minutes. In the majority of measurements, average signal intensity decreased on postcontrast STIR images compared to the corresponding precontrast STIR images. We assume that this is a reproducible technique.

It was previously shown in vitro that the signal intensity on STIR images is highly dependent on the gadolinium-based contrast agent concentration; e.g., concentrations between approximately 0.5 and 1.0 mmol per liter result in very low or even neutralized signal intensity on STIR images [3, 4]. This may explain in part the observed various degrees of absolute and relative signal loss on postcontrast STIR images compared to precontrast STIR images in bone marrow edema-like signal in the present study. One may speculate that various concentrations of gadolinium-based contrast agent in the analyzed tissue are related to the various degree of signal loss on postcontrast STIR images compared to precontrast STIR images in bone marrow edema-like signal.

Postcontrast signal loss was significantly larger in bone marrow edema-like signal than in healthy tissue, proving that STIR suppresses signal intensity values that correspond to gadolinium containing tissue in the clinical routine setting of the present study. Contrast agent results in an unreliable detection of bone marrow edema-like signal and may even completely obscure pathology. This represents a potential pitfall in a clinical scenario: The reporting radiologist may not be aware that the analyzed STIR sequence was acquired after contrast agent administration; e.g., the STIR sequence was repeated after intravenous contrast administration because a prior, precontrast STIR sequence was impaired by motion artifacts. Therefore, pathological MRI findings may be missed due to gadolinium-based obscuring on STIR images.

### Limitations

Initially, 60 patients were included; however, the lack of bone marrow edema-like signal in the Lisfranc joint accounted for exclusion of almost one-half (n = 29) of the patients which may weaken statistical conclusions. Furthermore, only the effect of gadoteric acid on STIR images was evaluated, but a similar effect from various types of contrast agents can be hypothesized.

## Conclusions

Although the STIR technique is generally considered a robust fluid-sensitive sequence, intravenous contrast agent administration causes a significant decrease of the signal intensity of bone marrow edema-like signal. This phenomenon of suppressed short T1 values of gadolinium containing tissue may obscure pathological MRI findings. STIR images should not be acquired after intravenous contrast agent administration if positive enhancement is the expected effect.
